# Comparison between airborne ultrasound and contact ultrasound to intensify air drying of blackberry: Heat and mass transfer simulation, energy consumption and quality evaluation

**DOI:** 10.1016/j.ultsonch.2020.105410

**Published:** 2020-12-06

**Authors:** Yang Tao, Dandan Li, Wai Siong Chai, Pau Loke Show, Xuhai Yang, Sivakumar Manickam, Guangjie Xie, Yongbin Han

**Affiliations:** a College of Food Science and Technology, Nanjing Agricultural University, Nanjing 210095, Jiangsu, China; b School of Chemical and Environmental Engineering, The University of Nottingham, Malaysia Campus, Semenyih, Selangor, Malaysia; c College of Mechanical and Electrical Engineering, Shihezi University, Shihezi 832000, China; d Petroleum and Chemical Engineering, Faculty of Engineering, Universiti Teknologi Brunei, Bandar Seri Begawan BE1410, Brunei Darussalam; e Zhihai Postgraduate Working Station, Zhenjiang, Jiangsu 212000, China

**Keywords:** Drying, Contact ultrasound, Airborne ultrasound, Heat and mass transfer, Blackberry

## Abstract

This study aimed at investigating the performances of air drying of blackberries assisted by airborne ultrasound and contact ultrasound. The drying experiments were conducted in a self-designed dryer coupled with a 20-kHz ultrasound probe. A numerical model for unsteady heat and mass transfer considering temperature dependent diffusivity, shrinkage pattern and input ultrasonic energies were applied to explore the drying mechanism, while the energy consumption and quality were analyzed experimentally. Generally, both airborne ultrasound and contact ultrasound accelerated the drying process, reduced the energy consumption and enhanced the retentions of blackberry anthocyanins and organic acids in comparison to air drying alone. At the same input ultrasound intensity level, blackberries received more ultrasound energies under contact sonication (0.299 W) than airborne sonication (0.245 W), thus avoiding the attenuation of ultrasonic energies by air. The modeling results revealed that contact ultrasound was more capable than airborne ultrasound to intensify the inner moisture diffusion and heat conduction, as well as surface exchange of heat and moisture with air. During air drying, contact ultrasound treatment eliminated the gradients of temperature and moisture inside blackberry easier than airborne ultrasound, leading to more homogenous distributions. Moreover, the total energy consumption under air drying with contact ultrasound assistance was 27.0% lower than that with airborne ultrasound assistance. Besides, blackberries dehydrated by contact ultrasound contained more anthocyanins and organic acids than those dried by airborne ultrasound, implying a higher quality. Overall, direct contact sonication can well benefit blackberry drying in both energy and quality aspects.

## Highlights

• Ultrasonic energy was input in both contact and airborne modes to dry blackberry.
• Ultrasonic influences on heat and mass transfer were simulated numerically.
• Shrinkage, input ultrasonic heat and temperature-dependent diffusivity were added.
• Contact sonication was more capable to accelerate drying and save energy.
• Contact sonication better preserved bioactives and flavors in blackberry.

## Nomenclature

*A*: surface area of blackberry fruit (m^2^)
*A*_540_: absorbance at 540 nm
*AAD*: absolute average deviation (%)
*A_Cu_*: surface area of copper sphere (m^2^)
*a_w_*: water activity
*C*: a constant in the GAB model shown in Eq. (1)
*C_air_*: concentration of water vapor in the air (kg m^−3^)
*C_p_*: specific heat (J kg^−1^ K^−1^)
*C_p,air_*: specific heat of air (J kg^−1^ K^−1^)
*C_p,Cu_*: specific heat of copper sphere (J kg^−1^ K^−1^)
*C_s_*: concentration of water vapor at blackberry surface (kg m^−3^)
*C_TA_*: total anthocyanin concentration expressed as mg/L of malvidin-3-glucoside equivalents (mg/L)
*d*: dilution
*D*_0_: pre-exponential factor of the Arrhenius equation (m^2^ s^−1^)
*D*_air_: diffusivity of water vapor in the air (m^2^ s^−1^)
*D_e_*: moisture effective diffusivity (m^2^ s^−1^)
*D_i,e_*: experimental data about temperature or moisture content (K or kg water/kg dry matter)
*D_i,p_*: predicted data about temperature or moisture content (K or kg water/kg dry matter)
*D_mean_*: mean value of the experimental data about temperature or moisture content (K or kg water/kg dry matter)
*E_a_*: activation energy (kJ mol^−1^)
*h*: heat transfer coefficient (W m^−2^ K^−1^)
*h_fg_*: water latent heat of evaporation (J/kg)
*h_m_*: mass transfer coefficient (m s^−1^)
*K*: a constant of GAB model shown in Eq. (1)
*Le*: Lewis number
*M*_0_: initial weight of blackberry fruit (kg)
*M*: weight of blackberry fruit (kg)
*m_cu_*: weight of copper sphere (kg)
*n*: number of experimental data
*P_US_*: ultrasound power (W)
*p_vs_*: saturated water vapor pressure on blackberry surface or in the air (Pa)
*Q_e_*: internal heat source or sink (W m^−3^, considered to be 0)
*Q_US_*: ultrasound intensity dissipated on blackberry surface in the form of heat (W m^−2^)
*r*: radius of whole blackberry fruit or blackberry sphere (m)
*R*^2^: coefficient of determination
*R_g_*: gas constant (kJ mol^−1^ K^−1^)
*RMSE*: root mean square error (K or kg water/kg dry matter)
*RH*: relative humidity of air
*t*: time (s)
*T*: temperature (K or ^o^C)
*T*_0_: initial temperature of blackberry or copper sphere (K or ^o^C)
*T_air_*: air temperature (K or ^o^C)
*T_Cu_*: surface temperature of copper sphere (K or ^o^C)
*T_s_*: surface temperature of blackberry (K or ^o^C)
*V_bl_*: volume of blackberry fruit (m^3^)
*V_Cu_*: volume of copper sphere (m^3^)
*W*: moisture content (kg water/kg DM)
*W*_0_: initial moisture content (kg water/kg DM)
*W_eq_*: moisture content at equilibrium (kg water/kg DM)
*W_m_*: monolayer moisture content in Eq. (1) (kg water/kg DM)
*X*: axial coordinate (m)

**Greek symbols**
*α_air_*: thermal diffusivity of air (m^2^ s^−1^)
*λ*: thermal conductivity (W m^−1^ K^−1^)
*ρ_air_*: air density (kg m^−3^)
*ρ_b_*: bulk density of blackberry (kg m^−3^)
*ρ_Cu_*: copper density (kg m^−3^)
*ρ_s_*: density of solid product (kg m^−3^)

## 1 Introduction

Dehydration is an old but effective technology for the preservation of perishable foods like fruits and vegetables. In today’s food industry, convective hot-air drying is the most widely applied dehydration method. However, the deficiencies of convective air drying are obvious, including high energy consumption, deterioration of food quality, loss of food nutrition, and relatively low drying rate [1], [2]. On the other hand, with the increase of energy cost and consumers’ demand for nutritive foods, a lot of efforts have been made to replace or improve convective drying technology, like freeze drying, microwave drying, heat pump drying, ultrasound drying, infrared drying, and hybrid drying technologies.

As a representative non-thermal technology, ultrasound has attracted worldwide interest in food processing field due to its capacity to benefit food processes. The report about the first ultrasound application in drying area dated back to the 1950 s, and the number of studies on ultrasound-assisted food drying has increased rapidly in the last two decades [3]. A consensus on ultrasound-assisted food drying has be achieved that mechanical vibration and cavitation phenomenon generated by ultrasound can speed up the drying process, reduce energy consumption and enhance the quality of dehydrated foods [3], [4], [5]. Due to these merits, ultrasound has a big potential to be applied in food drying industry. There are three different modes for ultrasound application in drying, involving ultrasound pretreatment, airborne or contactless ultrasound, and contact ultrasound. In practical applications, airborne ultrasound and contact ultrasound are usually incorporated into the dryer, thus intensifying the drying process directly [6], [7], [8]. The major difference between airborne ultrasound and contact ultrasound lies in the way acoustic energies reach food materials. For airborne ultrasound, a distance is kept between ultrasound vibrator and food materials, whereas foods can contact the sonotrode in the case of contact ultrasound application. Theoretically, contact ultrasound can conquer the problems of high attenuation of acoustic energy by air and acoustic impedance mismatch between food and air occurred during airborne ultrasound application, making contact ultrasound more effective and attractive [3], [9], [10]. Judging from the existing literature, the works on utilization of airborne ultrasound and contact ultrasound for food drying are mostly conducted separately, with a lack of data comparing the performance of both technologies. To promote an effective use of ultrasound in food industry, it is of paramount importance to conduct a comparative research between airborne ultrasound and contact ultrasound in the aspects of drying mechanism and food quality.

Drying is a complicated process involving simultaneous heat and mass transfer. To achieve a better understanding and control of drying process, it is necessary to know the spatial and temporal variations of temperature and moisture inside food materials. Mathematical simulation using physical-based models is an important tool to explore the underlying mechanisms [2]. The physical models about drying consist of partial differential equations (PDEs) describing the physics of heat, mass and momentum transfer in food and air domains, and some operations are usually implemented to reduce the complexity of PDEs [11], [12]. In the literature, researchers from Spain and China extensively simulated moisture transfer under ultrasound-assisted drying considering internal and external mass transfer resistances and volume shrinkage [6], [9], [13], [14], [15]. Meanwhile, Polish scientists used ordinary differential equations to model the temporal variations of temperature and moisture in food materials under airborne ultrasound assisted drying [16], [17], [18]. However, the current modeling studies on ultrasound-assisted drying could not fully represent the drying process. Mass transfer model alone usually ignores the influence of temperature variation on moisture effective diffusivity and assumes a constant value, whereas actual diffusivity varies with temperature [19], [20]. The ordinary differential equations cannot provide the information about spatial variations of temperature and moisture, and the influences of temperature on thermodynamic properties of food materials and drying medium are not included [12], [20], [21]. To enrich the fundamental knowledge about drying mechanism under the assistance of ultrasonic energies in both airborne and contact modes, coupled heat and mass transfer physical models considering temperature dependent diffusivity, volume shrinkage and input ultrasonic energies are needed.

As a recognized healthy fruit, blackberry is a rich source of natural antioxidants like polyphenols [22], [23]. Fresh blackberries are exceptionally perishable due to high moisture content and soft skin. Drying is an important way for the disposal of blackberry and extension of its shelf life. Dried blackberries are nutritious snacks with chewy texture, sweet and tart flavors. Apart from being eaten straight out of bag, dried blackberries can also be a gorgeous addition to other foods like salads and baked goods. Therefore, the demand for dried blackberry in the market is increasing continuously. The common way for dehydration of blackberry is air drying. However, bioactives in blackberry, particularly anthocyanins, are liable to be degraded under the exposure to hot air [24]. Thus, it is of interest to use ultrasound technology to accelerate air drying of blackberry and protect the bioactives. To date, ultrasound has been merely used as a pretreatment for air drying of blackberry [22], [25], whereas neither airborne ultrasound nor contact ultrasound has been applied for blackberry dehydration.

The objective of this paper is to compare the performance of airborne ultrasound and contact ultrasound in intensifying air drying of local blackberry (*Rubus americanus Britton*). Coupled heat and mass transfer models considering the factors of moisture diffusivity variation, shrinkage and input ultrasound energies were employed to depict the drying mechanism under the irradiation of ultrasound energies. Furthermore, energy consumption and changes of bioactives like phenolics and flavor compounds including organic acid were also analyzed. All these researches are expected to broaden the applicability of ultrasound technology in food drying area.

## 2 Materials and methods

### 2.1 Blackberry

Fresh blackberry fruits (*Rubus americanus Britton*, cultivar: *Hull*) were purchased from the plantation in Lishui, Nanjing, which affiliated to Institute of Botany, Jiangsu Province and Chinese Academy of Science. Considering that blackberry was perishable and fresh blackberries could not always be provided throughout the entire research, the samples were stored at −20 °C prior to experiments. Before each experiment, blackberries were thawed at room temperature and excessive liquids on blackberry surface were gently removed using tissue papers.

### 2.2 Drying treatment

Drying treatment was performed in a self-assembly hybrid dryer involving ultrasound irradiation and hot air flow inside. An ultrasound probe (20 kHz) with a diameter of 5 cm was inserted into the oven from the top. There was a sample holder underneath the ultrasound probe. Blackberry samples (approximately 3.5 g) were placed on the sample holder during drying. Through adjusting the height of sample holder, sonication can switch between airborne mode and contact mode. Hot air at target temperature entered the dryer from the left side and exited from the opposite side. The actual power delivered from ultrasound probe was measured using the calorimetric method by measuring the temperature rising of distilled water in a 100-mL glass beaker insulated by cotton cloth [26]. The ultrasound power was then transformed to ultrasound intensity, which was expressed as W/dm^2^.

**Fig. 1:**
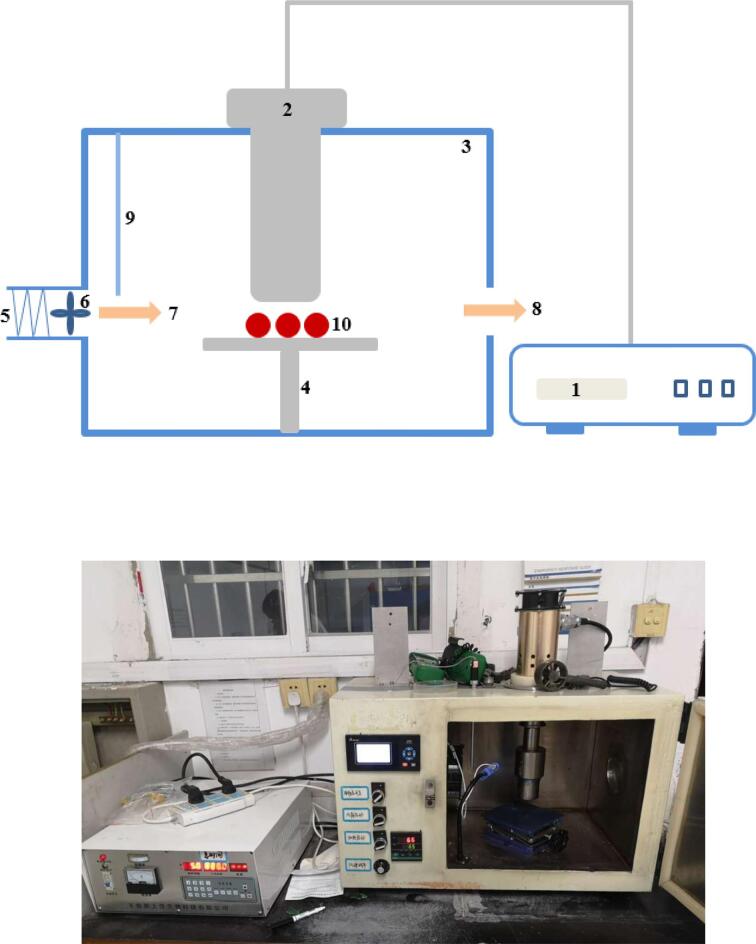
Experimental setup for ultrasound-assisted air drying of blackberry. 1: ultrasound generator; 2: ultrasound probe; 3: air dryer; 4: sample holder; 5: electrical heater; 6: electrical fan; 7: air inlet; 8: air outlet; 9: thermometer; 10: blackberry.

Blackberry fruits with similar size were selected for drying experiment. Blackberry fruits were placed on the sample holder. The actual output ultrasound intensity of the device was set at 180.1 W/dm^2^. The air temperature and velocity from the air inlet were 65 °C and 2.0 m/s. For surface-contact sonication, the ultrasound probe touched blackberry samples gently. Considering the shrinkage phenomenon, the height of sample holder was adjusted periodically, ensuring the direct contact between blackberry and ultrasound probe throughout the drying process. For airborne sonication, blackberry samples were placed 1 cm underneath the ultrasound probe. The weight of blackberry fruits was monitored until a constant value was reached. Hot-air drying without any sonication was also performed as control treatment. The experimental setup is depicted in Fig. 1. All the treatments were replicated for three times.

### 2.3 Shrinkage determination

The shrinkage pattern of blackberry was determined following the method of Tao et al. [13]. Specifically, blackberries were dried at 65 °C in both cases with and without sonication. Moisture content and fruit size were measured at different stages of drying. Since blackberries could maintain their spherical geometry despite of the moisture loss (Supplementary Fig. 1), a quantitative relationship between moisture ratio and sample radius ratio was built, which expressed the change of sample radius ratio as a function of moisture ratio.

### 2.4 Water sorption isotherm

Water sorption isotherm of blackberry was built following the static-gravimetric method described by Tao et al. [13]. Fresh blackberries were first dried in hot-air dryer to remove most of the water content, milled and moved to different glass desiccators containing saturated salt solutions inside (LiCl, CH_3_COOK, MgCl_2_, K_2_CO_3_, KI, NaCl and KCl). A small amount of thymol was added to avoid the potential microbiological contamination. The desiccators were sealed tightly and placed in an air oven at 65 °C. The weight of blackberries was monitored periodically until a constant weight was achieved (approximately 40 days). Then, the moisture content of blackberry at equilibrium was determined. The Guggenheim-Anderson-De Boer (GAB) model was employed to build the sorption isotherm of blackberry at 65 °C:

(1) $$W_{\mathrm{eq}}=\frac{W_{m}CKa_{w}}{\left(1-Ka_{w}\right)\left(1-Ka_{w}+CKa_{w}\right)}$$

### 2.5 Mathematical investigation

#### 2.5.1 Coupled heat and mass transfer modeling

The heat transfer modeling was based on the energy conservation law and Fourier’s law [27], while the mass transfer modeling was on the basis of Fick’s second law [19]. Prior to mathematical simulation, the following assumptions were made:

• Blackberry was considered as spherical geometry. There were no gradients of moisture and temperature at the centerline due to symmetry.
• Heat and mass transfer inside blackberry proceeded through conduction and diffusion, respectively.
• Phase change only occurred on blackberry surface.
• Initial moisture content, temperature and composition of blackberry were uniform.
• Heat and mass transfer coefficients stayed constant along blackberry surface.
• Volume shrinkage was accounted through the calculation of both *ρ*_b_ and *ρ*_s_, which referred to total mass and dry solid mass to total volume of blackberry fruit, respectively. Meanwhile, the path length of moisture diffusion and heat conduction inside blackberry that referred to sample radius also changed with volume shrinkage, resulting in the variation of boundary conditions [13]. The changes of radius of blackberry fruit was also involved in the heat and mass transfer models.

Hence, the governing equations of one-dimensional unsteady heat and mass transfer for spherical geometry in Cartesian coordinate are written as:

(2) $$\rho_{b}C_{p}\frac{\partial T}{\partial t}=\frac{1}{x^{2}}\frac{\partial }{\partial x}\left(x^{2}\lambda \frac{\partial T}{\partial x}\right)+Q_{e}$$

(3) $$\frac{\partial \left(\rho_{s}W\right)}{\partial t}=\frac{1}{x^{2}}\frac{\partial }{\partial x}\left(x^{2}\rho_{s}D_{e}\frac{\partial W}{\partial x}\right)$$

In the case of convective drying enhanced by ultrasound, the internal heat source or sink *Q*_e_ was considered to be 0, since ultrasonic energies were input from the external environment [27], [28].

The initial conditions are written as:

(4) $$T\left(x,t=0\right)=T_{0}$$

(5) $$W\left(x,t=0\right)=W_{0}$$

The boundary conditions are expressed as:

(6) $${\left(\frac{\partial T}{\partial x}\right|}_{x=0}=0$$

(7) $${\left(\frac{\partial W}{\partial x}\right|}_{x=0}=0$$

(8) $${\left(-\lambda \frac{\partial T}{\partial x}\right|}_{x=r}=h\left(T_{s}-T_{\mathrm{air}}\right)-h_{\mathrm{fg}}D_{e}\rho_{s}{\left(\frac{\partial W}{\partial x}\right|}_{x=r}+Q_{\mathrm{US}}$$

(9) $${\left({\rho_{s}D}_{e}\frac{\partial W}{\partial x}\right|}_{x=r}=h_{m}\left(C_{s}-C_{\mathrm{air}}\right)$$

Eq. (8) indicates that the heat transfer velocity due to conduction at blackberry surface was equal to the convective heat transfer from hot air to blackberry surface, heat transfer due to moisture vaporization, and heat transfer due to ultrasound irradiation. Eq. (9) represents that moisture migration to blackberry surface through diffusion was equal to moisture transfer by convection from the surface to the air.

The concentrations of vapor on sample surface and in the air are calculated using the ideal gas law (Eqs. (10), (11)) [19], [20]:

(10) $$C_{s}=2.1667\times 10^{-3}\times a_{w}\times \frac{p_{\mathrm{vs}}\left(T\right)}{T}$$

(11) $$C_{\mathrm{air}}=2.1667\times 10^{-3}\times \frac{\mathrm{RH}}{100}\times \frac{p_{\mathrm{vs}}\left(T_{\mathrm{air}}\right)}{T_{\mathrm{air}}}$$

Herein, the value of *a_w_* on sample surface was determined by the sorption isotherm relationship, while the water vapor pressure was calculated using Eq. (12) [12, 29]:

(12) $$p_{\mathrm{vs}}=exp\left[\frac{-5.8\times 10^{3}}{T}+1.391-4.864\times 10^{-2}T+4.176\times 10^{-5}T^{2}-1.445\times 10^{-8}T^{3}+6.545ln\left(T\right)\right]$$

Other thermophysical parameters, like specific heat *C_p_* [30], water latent heat of evaporation *h_fg_* [29] and thermal conductivity *λ* [31], are determined as follows:

(13) $$C_{p}=\left(0.837+1.256W\right)\times 1000$$

(14) $$h_{\mathrm{fg}}=2501.05\times 10^{3}\times {\left(\frac{647.3-T}{647.3-273.15}\right)}^{0.3298}$$

(15) $$\lambda =0.149+0.493\left(\frac{W}{1+W}\right)$$

Moreover, the moisture loss and volume shrinkage led to the decreases of both bulk density (*ρ_b_*) and density of solid product (*ρ_s_*). Since blackberry remained the spherical geometry throughout drying process (Supplementary Fig. 1) and the initial dry mass ratio in fresh blackberry was 13.8% determined according to the AOAC method [32], *ρ_s_* was calculated as:

(16) $$\rho_{s}=\frac{M_{0}\times 0.138}{V_{\mathrm{bl}}}=\frac{M_{0}\times 0.138}{\frac{4}{3}\pi r^{3}}$$

Moisture content in dry basis (*W*) was estimated as:

(17) $$W=\frac{M-M_{0}\times 0.138}{M_{0}\times 0.138}$$

After the transformation of Eq. (17), *M* was expressed as:

(18) $$M=W\times M_{0}\times 0.138+M_{0}\times 0.138$$

Thus, *ρ_b_* was represented as the following formula:

(19) $$\rho_{b}=\frac{M}{V_{\mathrm{bl}}}=\frac{W\times M_{0}\times 0.138+M_{0}\times 0.138}{\frac{4}{3}\pi r^{3}}$$

The coupled heat and mass transfer models were solved using the “*pdepe*” function in MATLAB. The number of mesh point in the radial direction was optimized, so as to minimize the *RMSE* value calculated using both temperature and moisture data. Another two indicators, namely *R*^2^ and *AAE* were also calculated to evaluate the predictive ability of the applied model. *R*^2^, *RMSE* and *AAE* are expressed as:

(20) $$R^{2}=1-\frac{\sum_{i=1}^{n}{\left(D_{i,p}-D_{i,e}\right)}^{2}}{\sum_{i=1}^{n}{\left(D_{i,e}-D_{\mathrm{mean}}\right)}^{2}}$$

(21) $$\mathrm{RMSE}=\sqrt{\frac{1}{n}\sum_{i=1}^{n}{\left(D_{i,p}-D_{i,e}\right)}^{2}}$$

(22) $$\mathrm{AAD}=\left[\frac{\sum_{i=1}^{n}\left(\left|D_{i,p}-D_{i,e}\right|/D_{i,e}\right)}{n}\right]\times 100$$

#### 2.5.2 Determination of effective moisture diffusivity

The *D_e_* value was computed using the analytical solution of Fick’s second law of diffusion for spherical geometry, which is a semi-empirical manner [33], [34]:

(23) $$\frac{W-W_{\mathrm{eq}}}{W_{0}-W_{\mathrm{eq}}}=\frac{6}{\pi^{2}}exp\left[-D_{e}{\left(\frac{\pi }{r}\right)}^{2}t\right]$$

Prior to using Eq. (23) for calculation, a series of assumptions were required, including constant *D_e_* value, uniform distributions of moisture and temperature and no external mass transfer resistance [35]. To satisfy these hypotheses, blackberry fruits were cut into small spheres with the diameter of merely 5 mm. Then, blackberry spheres were dried at different temperatures (50, 60 and 70 °C) with and without sonication, respectively. The air velocity was adjusted to the highest level (5 m/s), thus minimizing the external mass transfer resistance on blackberry surface. The sample weight was measured continuously until the drying process reached equilibrium.

The *D_e_* value was deduced from the slope of the straight line when plotting the logarithm of (*W*-*W_eq_*)/(*W_0_*-*W_eq_*) against time. Then, the influence of temperature on *D_e_* was explored using the Arrhenius-type temperature dependence equation:

(24) $$D_{e}=D_{0}exp\left(-\frac{E_{a}}{R_{g}T}\right)$$

#### 2.5.3 Determination of heat and mass transfer coefficients

In the literature, the Nusselt, Sherwood and Prandtl numbers for laminar and turbulence flows were usually employed to calculate the heat and mass coefficients of agriproducts undergoing drying [28], [36], [37]. However, the swirling of electric fan may lead to the inaccurate estimation [21], [38]. To integrate the influence of ultrasound irradiation on the heat and mass transfer coefficients, the approach reported by Pasban et al. [12] was used for the estimation of heat and mass transfer coefficients. For this purpose, a copper sphere with similar geometry of blackberry (25 mm in diameter) was placed in the drying oven at 65 °C under all the aforementioned drying conditions (non-sonication, airborne sonication, contact sonication). The surface temperature of copper sphere was recorded using a *K*-type thermocouple connected with a datalogger (DT-3891G, Shenzhen Everbest Machinery Industry Co. Ltd., China).

The lumped analytical method with the assumption of low internal temperature gradient can be applied to describe the heat transfer process on this copper sphere [21]:

(25) $$\frac{T_{\mathrm{Cu}}-T_{\mathrm{air}}}{T_{0}-T_{\mathrm{air}}}=\exp\left(\frac{-hA_{\mathrm{Cu}}}{\rho_{\mathrm{Cu}}C_{p,Cu}V_{\mathrm{Cu}}}t\right)$$

The *h* value was calculated through non-linear fitting of the data into Eq. (25) in MATLAB. The density of copper *ρ_Cu_* is 8.96 × 10^3^ kg m^−3^, and the specific heat of copper *C_p,Cu_* is 390 J kg^−1^ K^−1^ [39]. Then, the mass transfer coefficient *h_m_* was determined using the Chilton-Colburn analogy [12], [21]:

(26) $$h_{m}=\frac{h}{\rho_{\mathrm{air}}C_{p,air}{\mathrm{Le}}^{2/3}}$$

where the values of *ρ_air_* and *C_p,air_* are taken as 1.029 kg m^−3^ and 1.0 × 10^3^ J kg^−1^ K^−1^ [21]. The *Le* number was calculated as:

(27) $$\mathrm{Le}=\frac{\alpha_{\mathrm{air}}}{D_{\mathrm{air}}}$$

where the values of *α_air_* and *D_air_* are 2.5 × 10^-5^ m^2^ s^−1^ and 2.18 × 10^-5^ m^2^ s^−1^, respectively [21].

#### 2.5.4 Determination of actual ultrasound intensity dissipated on blackberry under airborne sonication and contact sonication

The actual ultrasound energy dissipated on blackberry fruits were determined using the calorimetric method [26]. Due to the lack of specific heat value for blackberry fruit, a copper sphere with similar geometry of blackberry (25 mm in diameter) was utilized for the measurement. Specifically, copper sphere was insulated using paper box. The temperature rising on ball surface under both airborne sonication and contact sonication at the output intensity of 180.1 W/dm^2^ from the ultrasound probe that was mentioned in Section 2.2 were monitored. The experimental setup for the determination of ultrasound energy is plotted in Supplementary Fig. 2. Then, the ultrasonic power dissipated on copper sphere surface was obtained from the following equation, which was regarded equal to the actual ultrasound power on blackberry surface under the applied drying conditions:

(28) $$P_{\mathrm{US}}=m_{\mathrm{Cu}}C_{p,Cu}\frac{\mathrm{dT}}{\mathrm{dt}}$$

Herein, the actual powers for airborne sonication and contact sonication were 0.245 and 0.299 W, respectively.

Accordingly, the ultrasound intensity was calculated as:

(29) $$Q_{\mathrm{US}}=\frac{P_{\mathrm{US}}}{A}$$

*Q_us_* changed with the decrease of blackberry surface due to volume shrinkage, which was determined at each sampling time on the basis of shrinkage pattern formula.

In summary, the flow chart for numerical simulation is illustrated in Supplementary Fig. 3.

### 2.6 Energy consumption determination

The amount of electricity consumed under each drying treatment was measured by an electricity meter (OKELE, Wenzhou, China).

### 2.7 Physicochemical analysis

In this section, blackberries were also dried in a freeze dryer (Freezezone 4.5, Labconoco, USA) for 48 h, in order to compare freeze-dried samples with air dried samples in quality aspects.

#### 2.7.1 Extraction of families of phenolics and organic acids

Phenolic compounds and organic acids in blackberries were extracted using the method similar to that described in our previous study [40]. Aqueous ethanol was selected as solvent for extraction, since both phenolics and organic acids dissolved well inside. First, dried blackberries were ground into powders using a grinder (All basic S25, IKA, Guangzhou, China). Then, blackberry samples were mixed with 50% aqueous ethanol at a solvent-to-solid ratio of 30:1 (mL:g). The extraction process was conducted at 30 °C under orbital agitation at 150 rpm for 2 h. After that, the supernatant was collected through centrifugation at 8000 rpm for 10 min. The residual phenolics and organic acids in blackberry samples were further extracted using fresh 50% aqueous ethanol. The extraction treatments were repeated in triplicates and the three supernatants were integrated. The crude extracts were stored at 4 °C in darkness before further analysis.

#### 2.7.2 Contents of total phenolics and total anthocyanins

Total phenolic content was measured using the Folin-Ciocalteu method [41]. To be exact, 1.0 mL of the phenolic extract with proper dilution was mixed with 1.0 mL of 10-fold-diluted Folin-Ciocalteu reagent, 3 mL of 7.5% sodium carbonate solution and 5.0 mL of distilled water. The mixture was vortexed and stayed at room temperature for 1 h. The absorbance at 765 nm was then detected in a UV–Visible spectrophotometer (Alpha-1500, Lab-Spectrum Instruments Co., Ltd, Shanghai, China). The result was expressed as mg gallic acid equivalents /g DW.

Total anthocyanin content was measured using the spectrophotometric method of Ivanova et al. [42]. The phenolic extract was diluted by a solution containing ethanol:water:HCl = 69:30:1 (v:v:v) properly. The absorbance at 540 nm was measured in the same spectrophotometer. Total anthocyanin concentration was calculated as:

(30) $$C_{\mathrm{TA}}=A_{540}\times d\times 16.7$$

The results were expressed as malvidin-3-glucoside equivalents/ g DW.

#### 2.7.3 Contents of individual anthocyanins

Individual anthocyanins in blackberry were first identified by HPLC-MS/MS following the method of Tao et al. [40]. Then, the contents of individual anthocyaninic compounds were determined following the HPLC method of Cui et al. [43]. The analysis was performed in a Shimadzu HPLC system (LC-2010A, Shimadzu Corporation, Japan) coupled with an Agilent TC-C18 column (250 × 4.6 mm, 5 μm) was used. The mobile phases included trifluoroacetic acid (0.5%, A) and methanol (B). The chromatographic conditions were: 0–5 min, 90%-88% A; 5–14 min, 88%-87% A; 14–16 min, 87%-86% A; 16–18 min, 86%-84%; 18–19 min, 84%-82% A; 19–22 min, 82%-78% A; 22–35 min, 78%-70% A. The flow rate was 0.8 mL/min. The detection wavelength was 520 nm, and the column temperature was set at 30 °C. The injection volume was 20 μL. The standard of each individual anthocyanin was used to construct the calibration curve. The results were expressed as μg each anthocyanin/g DM.

#### 2.7.4 Contents of organic acids

Contents of organic acids in blackberry were also measured in the same Shimadzu HPLC system connected with the Agilent TC-C18 column, according to the method of Lima et al. [44]. The mobile phase was mono-potassium phosphate solution (0.08 M, pH 2.9). The flowing rate was 0.7 mL/min. The detection wavelength was 210 nm. The column temperature was 30 °C. The injection volume was 20 μL. The calibration curves were made using the standard organic acids. The results were expressed as μg each organic acid/g DM.

#### 2.7.5 Antioxidant ability *in vitro*

The antioxidant capacity of blackberry *in vitro* was assessed using the 2, 20 -azinobis-(3-ethylbenzothiazoline)-6-sulfonic (ABTS) and ferric reducing antioxidant power (FRAP) methods. Both methods were well-described in our previous study [45]. For ABTS assay, the ABTS radical cation (ABTṠ^+^) was prepared by reacting 10 mL of 7 mmol/L ABTS solution with 10 mL of 2.45 mmol/L potassium persulfate solution in darkness at room temperature for 16 h. Then, appropriate dilution was performed using 0.2 mol/L phosphate buffer (pH 7.4) to make the absorbance of ABTṠ^+^ solution at 734 nm 0.70 ± 0.02. Then, 0.2 mL of phenolic extract after proper dilution was mixed with 3.8 mL of the ABTṠ^+^ solution. The solution was vortexed and stayed at room temperature in darkness for 7 min. The absorbance at 734 nm was measured and the ABTS radical scavenging capacity was calculated. Trolox was applied as the standard to make the calibration curve. The results were expressed as μmol Trolox/g DM.

For FRAP assay, the FRAP reagent was freshly produced by mixing 10 mmol/L 2,4,6-Tripyridyl-s-Triazine solution in the solution comprising 40 mmol/L HCl, 300 mmol/L acetate buffer with pH at 3.6 and 20 mmol/L FeCl_3_·6H_2_O at a volume ratio of 1:10:1. Then, 0.1 mL of phenolic extract after proper dilution, 0.3 mL of deionised water and 3 mL of FRAP reagent were mixed together. The solution was vortexed and stayed at 37 °C in darkness for 8 min. The absorbance at 593 nm was detected. FeSO_4_ was applied to make the calibration curve and the results were expressed as μmol FeSO_4_/g DM.

### 2.8 Statistical analysis

One-way analysis of variance (ANOVA) was conducted in SPSS 11.5 (SPSS Inc., USA) to compare the means of quality data of blackberry under different treatments. Least Significant Difference (Fisher's LSD) test was applied and the significance was selected at *p* < 0.05.

## 3 Results and discussion

### 3.1 Moisture and temperature variations in blackberry during drying

**Fig. 2:**
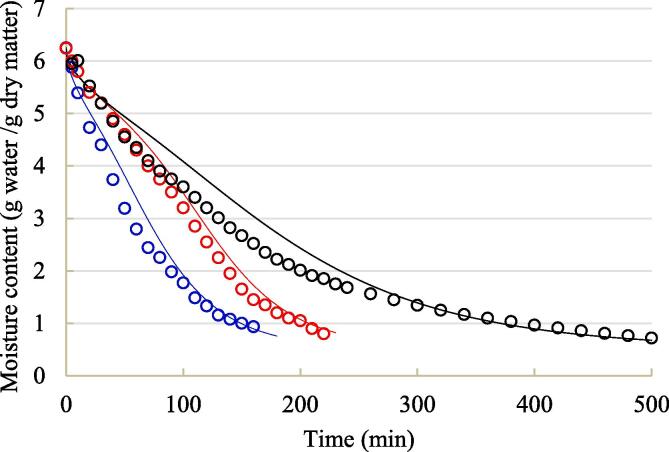
Average moisture content profile throughout hot-air drying with and without ultrasound assistance. o: air drying alone (black color); o: air drying coupled with airborne sonication (red color); o: air drying coupled with contact sonication (blue color). The solid line refers to modeling results.

**Fig. 3:**
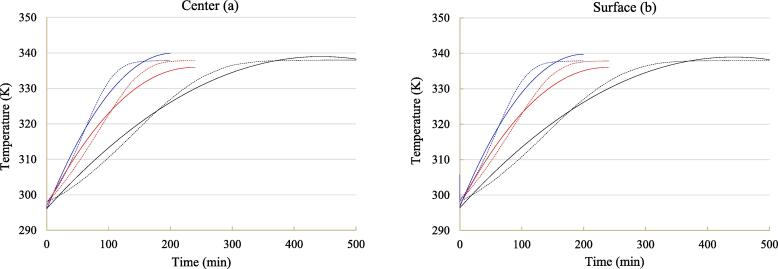
Evolutions of center (a) and surface (b) temperatures throughout hot-air drying with and without ultrasound assistance. Black line: air drying alone; red line: air drying coupled with airborne sonication; blue line: air drying coupled with contact sonication. The solid line denotes experimental data and dashed line denotes simulated data. (For interpretation of the references to colour in this figure legend, the reader is referred to the web version of this article.)

Drying kinetic curve and temperature history of blackberry are plotted in Fig. 2, Fig. 3, respectively. Unsurprisingly, moisture content in blackberry during contact ultrasound assisted drying declined the fastest, followed by airborne ultrasound drying and air drying alone. These results were in line with many studies on ultrasound drying reported in the literature [7], [9]. Meanwhile, the calorimetric determination in Section 2.5.4 also showed that under the same ultrasound intensity, the actual ultrasonic power dissipated on each blackberry under contact sonication (0.299 W) was 22.0% higher than that under airborne sonication (0.245 W). These results can prove that contact ultrasound can conquer the problems of acoustic energy attenuation by air and acoustic impedance mismatch between blackberry and air, thus possessing higher drying capacity than airborne ultrasound. It took approximately 150 min for air drying coupled with contact sonication, 205 min for air drying coupled with airborne ultrasound, and 390 min for air drying alone to reduce the moisture content to 1.0 g/g DW.

Referring to temperature profile, both surface and center temperatures of blackberry with and without sonication increased gradually close to air temperature during drying. Ultrasound treatment significantly intensified the temperature increasing rate, confirming that both heat convection on blackberry-air interface and heat conduction insider blackberry were improved by ultrasound. This phenomenon can be attributed to the vibration effect of ultrasound and adsorption of ultrasonic energy by blackberry [16], [46]. In comparison to airborne ultrasound, contact ultrasound was more effective to accelerate the heat transfer process under air drying. Moreover, the difference between surface temperature of sonicated blackberry and hot air temperature at the end of drying was in a small scale. This result implies that heating effect of both surface-contact ultrasound and airborne ultrasound did not lead to the overheating problem, which was in line with the studies of Szadzińska et al. [18] and Tao et al. [9]. Besides, the difference between surface temperature and center temperature for all the treatments was negligible, indicating a relatively uniform distribution of temperature within blackberry fruits. Homogenous distribution of temperature within food materials was also observed in air drying of longan [47].

### 3.2 Heat and mass transfer study

#### 3.2.1 Shrinkage pattern and water sorption isotherm

**Fig. 4:**
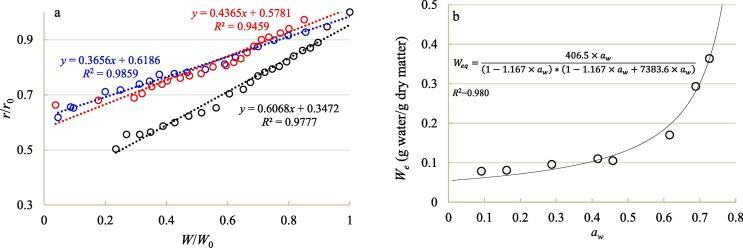
Shrinkage pattern of blackberry fruits under hot-air drying at 65 °C with and without sonication (a) and water sorption isotherm of blackberry fruit at 65 °C (b). Black circle and line in Fig. 4a: air drying alone; red circle and line in Fig. 4a: air drying coupled with airborne sonication; blue circle and line in Fig. 4a: air drying coupled with contact sonication. (For interpretation of the references to colour in this figure legend, the reader is referred to the web version of this article.)

Shrinkage pattern plays an important role for the determination of heat and mass transfer path length during numerical simulation [48]. The variation of blackberry size ratio as a function of moisture ratio is illustrated in Fig. 4a. It can be seen that the dimensionless size of blackberry decreased linearly with the decline of moisture ratio during drying, which was similar to the shrinkage patterns of many fruits and vegetables, like garlic, cabbage and apple [9], [13], [48]. The slopes of the shrinkage pattern equation for contact ultrasound treated samples, airborne ultrasound treated samples and non-ultrasound treated samples were 0.3656, 0.4365 and 0.6068, respectively. These values imply that both contact ultrasound and airborne ultrasound were able to alleviate the blackberry structure collapse during drying, while contact ultrasound was more effective to inhibit the volume deformation.

Moreover, the relationship between water activity and equilibrium moisture content at 65 °C was successfully fitted to the GAB model as shown in Eq. (1) (Fig. 4b), which was then used for roughly calculating the concentrations of vapor on sample surface in modeling. Herein, it should be explained that although the utilization of milled blackberry samples after partial dehydration for the construction of water sorption isotherm as mentioned in Section 2.4 may lead to the over-estimation problem, the time required to make the sample equilibrated in the static-gravimetric method was shortened markedly.

#### 3.2.2 Temperature-dependent moisture effective diffusivity

Effective moisture diffusivity *D_e_* is needed to solve the heat and mass transfer models as shown in Eqs. (3), (8) and (9). Since the information about *D_e_* under hot-air drying of blackberry is scarce in the literature, *D_e_* values at different temperatures was estimated using blackberry sphere with the diameter of 5 mm as a model material. The utilization of blackberry sphere with small size can help the drying kinetic data better match the assumptions of analytical solution of Fick’s second law, because the reduction of blackberry radius can accelerate the drying rate and achieve the uniform distributions of temperature and moisture more easily [49]. The drying kinetic curves of blackberry sphere at 50, 60 and 70 °C with and without sonication are plotted in Supplementary Fig. 4. The major findings exhibited in Supplementary Fig. 4 also demonstrated that ultrasound facilitated the interior moisture diffusion and consequently shortened the drying time of blackberry sphere, which were consistent with the results exhibited in Section 3.1. Based on Table 1, the Arrhenius relationship was capable to describe the change of *D_e_* as a function of temperature, which was then sent to the heat and mass transfer models.

**Table 1:** Arrhenius-type temperature dependence equations for blackberry moisture effective diffusivity under hot-air drying with and without sonication.

Treatment	Arrhenius-type temperature dependence equation
Air drying alone	$D_{e}\left(m^{2}\min^{-1}\right)=2.608\times 10^{-6}\times exp\left(-\frac{1159}{T}\right)$
Air drying coupled with airborne sonication	$D_{e}\left(m^{2}\min^{-1}\right)=0.007628\times \exp\left(-\frac{3562}{T}\right)$
Air drying coupled with contact sonication	$D_{e}\left(m^{2}\min^{-1}\right)=0.0000298\times \exp\left(-\frac{-1650}{T}\right)$

#### 3.2.3 Heat and mass transfer coefficients

The convective heat and mass transfer coefficients under airborne and contact ultrasonic fields with surrounded hot air are presented in Table 2. The calculated *h* and *h_m_* values ranged from 10.60 to 26.61 W m^−2^ K^−1^ and 0.01130 to 0.02833 m s^−1^, respectively, both of which were in the similar magnitude of the counterparts during drying of cylindrical quince slice, apple slice, cylindrical potato [12], [20], [50]. The *h* and *h_m_* values under contact sonication were the highest, followed by that under airborne sonication and non-sonication, indicating that contact ultrasound was more effective than airborne ultrasound to promote the heat and moisture exchanges with air on blackberry surface and consequently intensify the drying kinetics. Additionally, *h* and *h_m_* were considered constant during the whole drying process, assuming the same exposure of surface boundaries to hot air during modeling [19].

**Table 2:** Convective heat and mass transfer coefficients on blackberry surface at 65 °C.

Treatment	*h* (W m^−2^ K^−1^)	*h_m_* (m s^−1^)
Air drying alone	10.61	0.01130
Air drying coupled with airborne sonication	24.54	0.02613
Air drying coupled with contact sonication	26.61	0.02833

#### 3.2.4 Coupled heat and mass transfer modeling

With the aforementioned shrinkage pattern, sorption isotherm, temperature*-*dependent moisture diffusivity within blackberry, and heat and mass transfer coefficients on blackberry surface, the coupled heat and mass transfer models were successfully solved and the graphical comparisons are depicted in Figs. 2 and 3. In the meantime, the indices of fitness quality are summarized in Table 3. As can be seen, the applied models were able to predict the evolutions of temperature and moisture content during drying with acceptable accuracy, despite of several divergences in some areas. It should be pointed out that the number of grids made in radical direction of heat and mass transfer pathway markedly affected the predictive results of the applied models. Herein, blackberry radius was divided into 121 pieces when solving the partial differential equations using *pdepe* function. Moreover, a fluctuation of predicted surface temperature was observed in the very early stage of drying in Fig. 3b. The decline of temperature in this phenomenon can be attributed to the evaporation of water at blackberry surface [51].

**Table 3:** Predictive accuracy of the coupled heat and mass transfer models.

Model	Treatment	*R*^2^	*RMSE* (K or g water/g dry matter)	*AAD* (%)
Heat*	Air drying alone	0.992	1.513	0.42
	Air drying coupled with airborne sonication	0.983	1.755	0.47
	Air drying coupled with contact sonication	0.982	1.810	0.46
				
Mass	Air drying alone	0.978	0.341	11.12
	Air drying coupled with airborne sonication	0.990	0.241	9.50
	Air drying coupled with contact sonication	0.983	0.315	9.04

*: *R*^2^, *RMSE* and *AAD* values for heat transfer model were calculated using the experimental and predicted data about surface temperature.

**Fig. 5:**
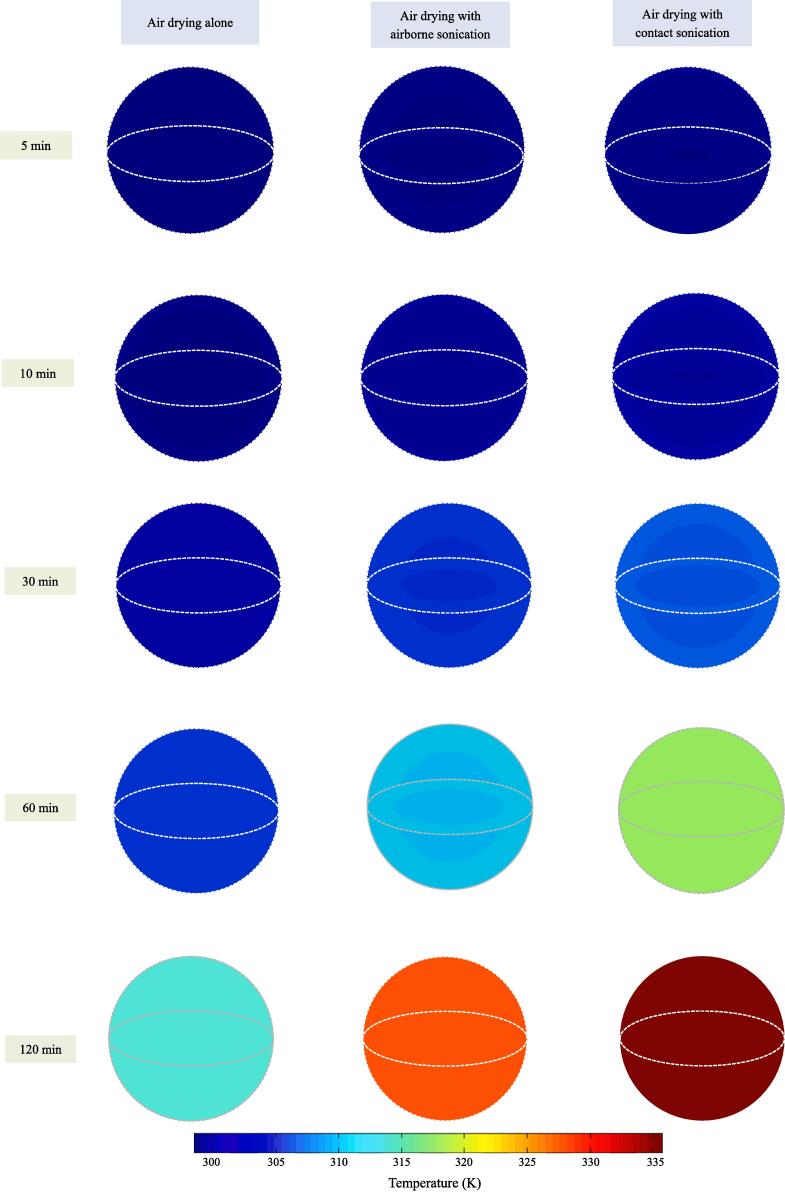
Spatial distribution of temperature within blackberry fruit under hot-air drying with and without ultrasonic assistance (The dashed line refers to the outer surface).

Following the successful numerical simulation, the spatial distributions of temperature within blackberry at several stages are illustrated in Fig. 5. As can be seen, the simulated temperature gradient within blackberry under air drying alone, air drying coupled with airborne sonication and air drying with contact ultrasonic assistance were all very small in the selected time periods. Meanwhile, the temperature inside blackberry fruit rose faster under ultrasonic treatment, especially contact sonication. The difference in temperature profile between blackberries with and without sonication became more obvious after 10-min air drying. After 120-min drying, the temperature of contact ultrasound treated blackberry reached close to the equilibrium temperature, and the temperature of airborne ultrasound treated blackberry also approached to air temperature. Generally, during the early stage of drying, the faster loss of moisture content in ultrasound-treated blackberries referred to the stronger evaporative cooling effect, which compensated the ultrasonic enhancement of heat exchange between blackberry surface and hot air. Consequently, the temperature difference between samples with and without sonication was less obvious. With the increase of drying time and decrease of moisture flux to blackberry surface, the higher heat transfer coefficient under sonication made the temperature of ultrasound-processed samples reach the equilibrium earlier [12]. All these results were well coincident with the experimental profiles of surface and center temperature under different treatments shown in Fig. 3, demonstrating that the coupled heat and mass transfer models with the applied thermodynamic parameters and hypotheses can inherently represent the actual temperature evolution.

**Fig. 6:**
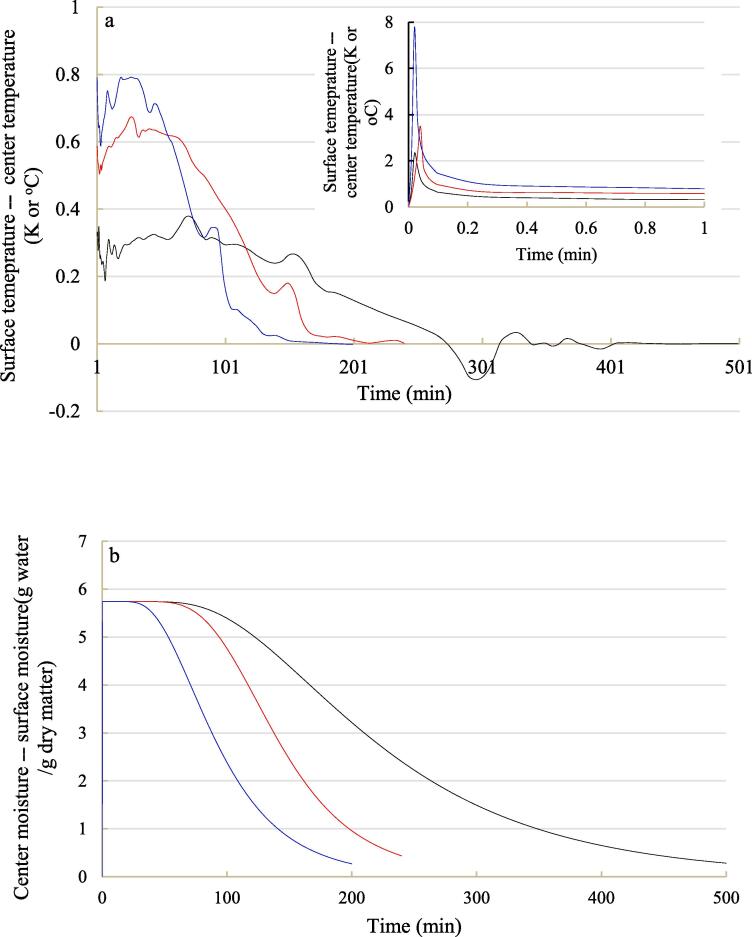
Temperature difference (a) and moisture content difference (b) between center and surface of blackberry during hot-air drying with and without sonication. Black line: air drying alone; red line: air drying with airborne sonication; blue line: air drying with contact sonication. (For interpretation of the references to colour in this figure legend, the reader is referred to the web version of this article.)

To gain a deeper physical insight about ultrasonic influence on heat transfer, the difference in temperature between surface and center was calculated and plotted on the basis of modeling results (Fig. 6a). Although the temperature difference between surface and center was small as shown in Fig. 3, Fig. 5, surface temperature still increased faster than center temperature, which was in coincidence with the reality. The difference between surface temperature and center temperature increased to the maximum merely in the first 2 s of drying, and then declined rapidly below 1 K within 15 s. Among the three applied drying methods, contact sonication created the largest temperature difference between blackberry surface and core, of which the value was close to 8 K. Meanwhile, the maximum temperature difference between surface and center under airborne ultrasound assisted air drying and only air drying were 3.5 K and 2.3 K, respectively. The relatively high surface heat transfer coefficient and input acoustic heat under contact sonication can facilitate the fast increase of surface temperature, thus making a big difference between surface temperature and core temperature at the beginning of drying. On the other hand, after the appearance of temperature difference peak, contact ultrasound treatment eliminated the temperature gradient inside blackberry faster than airborne ultrasound treatment and air drying alone. The direct contact between ultrasound vibrator and blackberry helped blackberry receive the strongest ultrasonic vibration, which can better improve the dissipation of heat [46].

**Fig. 7:**
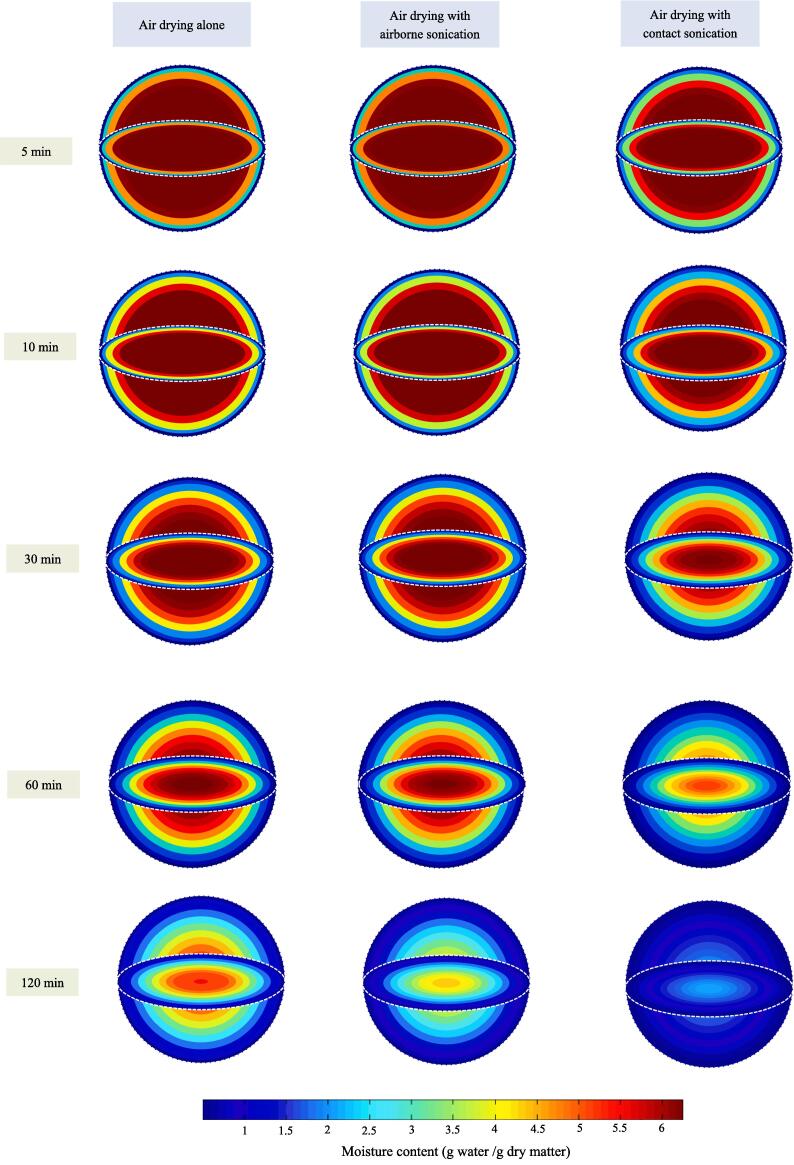
Spatial distribution of moisture content within blackberry fruit under hot-air drying with and without ultrasonic assistance (The dash line refers to the outer surface).

Unlike temperature distribution, inhomogeneous moisture distribution inside blackberries was observed at the early stage of drying, and the moisture gradient attenuated with the increase of drying time (Fig. 7). This phenomenon was common, since the outer surface surrounding hot air always got dried first and then the concentration gradient drove moisture move from the interior to the surface [20]. Meanwhile, the attenuation of moisture concentration gradient along with drying implied that the mass transfer driving force got weaken and the drying rate decreased due to water loss. The influences of contact and airborne sonication on moisture movement inside blackberry was also reflected by visualization of modeling results. The interior of blackberry treated by contact and airborne sonication got dried earlier than that without sonication. After drying for 120 min, moisture concentration gradient inside contact ultrasound treated blackberry almost diminished. The aforementioned high temperature rising rate contributed to the rapid attenuation of moisture concentration gradient under contact sonication, since the molecular diffusivity increased with temperature based on Einstein equation [52]. Moreover, the difference in moisture content between center and surface of blackberry during drying is plotted in Fig. 6b. It can be observed clearly that contact sonication diminished the difference fastest, followed by airborne sonication and only air drying.

Another interesting phenomenon about moisture transfer via simulation was that moisture content on the air-material interface for all the blackberry samples dried with and without ultrasonic assistance decreased from 6.25 to 0.51 g water /g dry matter instantaneously (<1 s) and then stayed constant under the exposure to hot air. This result implied that theoretically the drying process at air-blackberry interface reached equilibrium immediately when drying commenced under the selected conditions and this zone named “drying front” kept dry afterwards.

### 3.3 Energy consumption

The total energies required to reduce blackberry moisture content to 1.0 g/g DM were 2.67 kW h for air drying alone, 1.89 kW h for air drying with airborne ultrasound assistance, and 1.38 kW h for air drying with contact ultrasound assistance. Thus, the ultrasonic acceleration of heat and mass transfer can benefit the drying process through reducing the energy consumption, showing its potential to be applied in industrial drying as a green technology. Moreover, the energy consumption under contact sonication was 27.0% lower than that under airborne sonication treatment.

### 3.4 Quality evaluation

To further evaluate the benefits accompanied by the ultrasonic enhancement of heat and mass transfer during drying, the quality aspects, including contents of total phenolics, total anthocyanins, individual anthocyanins and organic acids, and antioxidant capacity *in vitro* were analyzed. For the comparison purpose, freeze-dried blackberries were chosen as a control.

According to Table 4, dehydrated blackberries receiving both contact sonication and airborne sonication possessed higher amounts of total phenolics and total anthocyanins than only air dried samples, and these differences between contact ultrasound-treated samples and air dried samples alone were statistically significant (*p* < 0.05).

**Table 4:** Comparison of quality indices among blackberries dehydrated by different methods.

Index	Freeze drying	Air drying alone	Air drying coupled with airborne sonication	Air drying coupled with contact sonication
Total phenolic content (mg gallic acid/g DM)	60.49 ± 2.03^ab^	58.08 ± 7.48^b^	62.14 ± 2.23^ab^	69.71 ± 2.56^a^
Total anthocyanin content (mg Cyaniding-3-*O*-glucoside/g DM)	6.57 ± 0.11^a^	5.47 ± 0.23^b^	5.62 ± 0.50^b^	6.66 ± 0.23^a^
Cyanidin-3-*O*-glucoside (μg/g DM)	5993.1 ± 223.8^a^	4622.5 ± 176.7^b^	5242.3 ± 347.4^b^	5935.8 ± 164.5^a^
Peonidin-3-*O*-glucoside (μg/g DM)	1201.9 ± 43.7^a^	726.6 ± 40.3^c^	901.0 ± 43.4^b^	1139.8 ± 37.6^a^
Malvidin-3-*O*-glucoside (μg/g DM)	148.6 ± 7.1^a^	84.7 ± 6.0^c^	115.0 ± 10.0^b^	146.5 ± 6.0^a^
Oxalic acid (μg/g DM)	1681.9 ± 49.9^a^	671.7 ± 30.4^d^	1002.5 ± 10.5^c^	1427.1 ± 9.2^b^
Malic acid (μg/g DM)	59201.6 ± 1441.8^a^	36001.2 ± 310.4^b^	33530.7 ± 657.2^c^	35972.2 ± 658.3^b^
Tartaric acid (μg/g DM)	8896.4 ± 358.8^a^	4755.1 ± 182.7^c^	5853.2 ± 829.9^b^	5114.9 ± 50.8^bc^
Citric acid (μg/g DM)	10562.7 ± 553.4^a^	4403.7 ± 615.7^b^	4058.8 ± 1141.5^b^	5639.8 ± 771.3^b^

ABTS radical cation scavenging activity (μmol Trolox/g DM)	2114.8 ± 35.2^a^	2328.7 ± 57.4^b^	2521.6 ± 24.9^c^	2610.4 ± 46.8^d^
Ferric reducing antioxidant power (μmol Fe^2+^/g DM)	0.935 ± 0.005^a^	0.962 ± 0.004^a^	1.031 ± 0.021^b^	1.055 ± 0.014^b^

Values followed by different letters in each line indicated significant differences (*p* < 0.05).

Total phenolic content and total anthocyanin content in contact ultrasound treated samples were 20.0% and 21.8% higher than that in only air-dried samples, respectively. Meanwhile, three individual anthocyanins were tentatively identified in the studied blackberry cultivar, including cyanidin-3-*O*-glucoside, peonidin-3-*O*-glucoside and malvidin-3-*O*-glucoside. These anthocyanins were also previously found in other blackberry cultivars [23], [53]. Contact ultrasound treated blackberries contained the highest contents of these individual anthocyanins among all the air-dried samples, followed by airborne ultrasound treated blackberries and only air-dried blackberries. For example, the contents of cyanidin-3-*O*-glucoside, peonidin-3-*O*-glucoside, malvidin-3-*O*-glucoside in dried blackberries treated with contact sonication were 413.2, 61.8 and 225.2 g/g DM higher than the counterparts in air-dried blackberries without sonication. In the literature, many studies have also reported that ultrasonication can provide protective effect on polyphenols during air drying of various food products. For example, Nascimento et al. [54] found that the phenolic retentions in dried passion fruit peel were enhanced by 54.6% and 49.5%, respectively, if sonication was performed under air drying at 40 °C and 50 °C. For ultrasound-assisted convective drying of carrots, sonication at 200 W significantly increased total phenolic content in dried carrots from 640 mg/kg DM to 719 mg/kg DM [55]. However, special attention should be paid that sonication cannot always protect food phenolic compounds under the exposure to hot air, or even intensify the destructive effect. Rojas et al. [56] found no significant difference in phenolic content between dehydrated apple slices with and without airborne sonication. The results of Santacatalina et al. [57] revealed that ultrasound application speeded up the degradation of phenolics during air drying of apple, probably due to ultrasonic assistance to the release of oxidative enzymes. Nascimento et al. [54] also revealed that ultrasound application and drying temperature had interaction effect on phenolic retention in the case of passion fruit peel, and sonication at relatively high temperature facilitated phenolic degradation. Thus, the processing parameters and characteristics of food materials can affect the retention of bioactives in dehydrated products treated by ultrasound.

Both ABTS radical cation scavenging activity and ferric reducing antioxidant power of the extracts from sonicated blackberries were significantly stronger than the extracts from air dried samples without sonication. At the same time, the antioxidant ability *in vitro* of contact ultrasound treated blackberry was higher than that of airborne ultrasound treated samples. To be exact, the ABTS radical cation scavenging activities for dried blackberries processed by contact sonication and airborne sonication were 12.1% and 8.3% higher than that for only air-dried samples. Meanwhile, the ferric reducing antioxidant powers for contact ultrasound and airborne ultrasound processed blackberries were 9.7% and 6.9% higher than that for air-dried blackberries without sonication. The stronger antioxidant capacity of ultrasound-treated blackberries corresponded to the aforementioned high amounts of phenolics including anthocyanins. Besides, the generation and accumulation of some Maillard reaction products and new phenolic compounds may also contribute to the higher antioxidant capacity of dried blackberries receiving ultrasound irradiation [54], [55], [58]. In the ultrasound-incorporated air drying area, Li et al. [59], Kroehnke et al. [55] and Fonteles et al. [60] found that ultrasound helped retain the antioxidant potential of hawthorn fruit juice, carrot and cashew apple bagasse puree, whereas ignorable or even negative effects of sonication on antioxidant capacity of dried products like apple and eggplant were reported by Santacatalina et al. [57] and Colucci et al. [61], owing to cell disruption caused by mechanical force of ultrasound.

On the other hand, the contents of total anthocyanins and three individual anthocyanins in only air-dried samples and airborne ultrasound-treated samples were all significantly (*p* < 0.05) lower than that in freeze-dried samples. However, contact ultrasound treated blackberries possessed significantly higher total phenolic content, comparable amounts of total anthocyanins and three individual anthocyanins and stronger antioxidant capacity in comparison to freeze-dried samples. These results indicate that contact ultrasound is a competitive technology to preserve nutrients in blackberries even compared with freeze drying technique. Generally, the ultrasonic enhancement of heat and mass transfer inside blackberry shortened its exposure to hot air, thus inhibiting thermal degradation of free phenolics in vacuoles, especially anthocyanins [62]. Apart from free phenolics, there are also a portion of phenolics bounded with cell wall [63]. It was possible that ultrasound treatment may affect the affinity between cell wall and bound phenolics or help the detachment of bound phenolics from cell wall, consequently increasing the retention of free phenolics in dried blackberry [64]. Moreover, the stability of phenolics during drying are also correlated with oxidative enzymes, such as polyphenoloxidases and peroxidases [55]. The rapid increase of temperature and loss of moisture in blackberry under sonication may accelerate the inactivation of oxidative enzymes, thus providing a protective effect on blackberry phenolics during air drying. Besides, the de novo synthesis of phenylalanine ammonia-lyase may also contribute to the accumulation of phenolics [55], [65]. Taking these discussions into consideration, the effects of both contact ultrasound and airborne ultrasound on the interaction between phenolics and cell wall, stability of oxidative enzyme and possible formation of phenolic derivatives during air drying deserve a deep investigation in future studies.

Organic acids are important flavor components in blackberry. Air drying resulted in an obvious loss of organic acids. The contents of all the individual organic acids in air-dried blackberries with and without ultrasound treatment were significantly lower than that in freeze-dried samples. In the study of Ojha et al. [66], the authors also found that drying led to significant losses of lactic acid and acetic acid in beef jerk. Meanwhile, the incorporation of sonication into air drying exhibited protective effects on the detected organic acids except malic acid. The contents of oxalic acid, tartaric acid and citric acid in dried blackberries with contact ultrasonic assistance were 1427.1 ± 9.2, 5114.9 ± 50.8, 5639.8 ± 771.3 μg/g DM, which were 755.4, 359.8 and 1236.1 μg/g DM higher than that in only air dried samples, respectively. At the same time, airborne ultrasound treated blackberries were also richer in oxalic acid and tartaric acid than air dried samples alone. The high retention of organic acids under ultrasound treatment can also be ascribed to the shorter exposure duration to hot air.

Overall, both nutritive and flavor components in blackberry can be protected in addition to ultrasonic enhancement of heat and mass transfer during air drying. Contact ultrasound was more capable than airborne ultrasound to retain phenolics and organic acids.

### 3.5 Thinking about design of ultrasound-incorporated air dryer

Currently, a big concern about the application of contact ultrasound in food drying is the scale-up of contact ultrasonic devices. In the past few years, our group has made many efforts to build ultrasound-assisted dryer with different designs [9], [10], and the developed ultrasonic vibrators are plotted in Supplementary Fig. 5. One key point from our experience is the type of metallic material used to make the ultrasound vibrator. Titanium alloy-made probes can work well with stable resonance with ultrasound transduce, since this material is robust enough to resist metal fatigue and deformation under long-time sonication. Aluminum alloy is another choice with lower cost, but aluminum alloy-made vibrator is more prone to metal fatigue under sonication. Another issue affecting the working performance of contact ultrasound equipment is the shape of ultrasound probe, although the underlying physics has not yet been well-understood. In future studies about designing and scaling up air dryer coupled with contact ultrasound, it is recommended to consider the influences of materials and shape of ultrasound vibrator, which allows the direct contact with food materials.

## 4 Conclusions

This study provided a straightforward comparison between airborne ultrasound and contact ultrasound to assist the air drying of blackberry. It was evident from the calorimetric determination that blackberries received more ultrasonic energies if they were in contact with ultrasonic probe directly, demonstrating that contact sonication successfully conquered the problems of ultrasonic energy attenuation and acoustic impedance mismatch between air and foods occurred under airborne sonication. The spatial and temporal evolutions of temperature and moisture content within blackberry were well depicted by numerical simulation taking the influence of temperature on moisture diffusivity, volume shrinkage and heat generated by ultrasound into account. Owing to the higher utilization of ultrasonic energies, the heat and mass transfer rates under contact sonication were higher than that under airborne sonication, and temperature and moisture content inside blackberries were also distributed more homogeneously. Together with a higher drying rate, less energies were required to dehydrate blackberries if ultrasonic energies were input in a contact way. Other advantages of contact sonication over airborne sonication were that contacted ultrasound-dried blackberries contained higher amounts of bioactives like anthocyanins and flavor compounds like organic acids than dried blackberries treated by airborne ultrasound. All the obtained results proved that contact ultrasound treatment can be a better choice to benefit the blackberry drying process.

## CRediT authorship contribution statement

**Yang Tao:** Conceptualization, Methodology, Investigation, Data curation, Software, Writing - original draft, Writing - review & editing. **Dandan Li:** Writing - review & editing. **Wai Siong Chai:** Writing - review & editing. **Pau Loke Show:** Writing - review & editing. **Xuhai Yang:** Writing - review & editing. **Sivakumar Manickam:** Writing - review & editing. **Guangjie Xie:** Writing - review & editing. **Yongbin Han:** Writing - review & editing, Supervision.

## Declaration of Competing Interest

The authors declare that they have no known competing financial interests or personal relationships that could have appeared to influence the work reported in this paper.
