# Supplementary material for: Comparison between airborne ultrasound and contact ultrasound to intensify air drying of blackberry: Heat and mass transfer simulation, energy consumption and quality evaluation
Source: Ultrason Sonochem. 2020 Dec 6;72:105410. doi: 10.1016/j.ultsonch.2020.105410 (PMC7803686; doi:10.1016/j.ultsonch.2020.105410)
Supplement: Supplementary data 1 [file mmc1.docx]

**Comparison between airborne ultrasound and contact ultrasound to intensify air drying of blackberry: Heat and mass transfer simulation, energy consumption and quality evaluation**

Yang Tao^a^, Dandan Li^a^, Wai Siong Chai^b^, Pau Loke Show^b^, Xuhai Yang^c^, Sivakumar Manickam^d^, Guangjie Xie^a, e^, Yongbin Han^a,^*

^a^ College of Food Science and Technology, Nanjing Agricultural University, Nanjing 210095, Jiangsu, China

^b^ School of Chemical and Environmental Engineering, The University of Nottingham, Malaysia Campus, Semenyih, Selangor, Malaysia

^c^ College of Mechanical and Electrical Engineering, Shihezi University, Shihezi 832000, China

^d^ Petroleum and Chemical Engineering, Faculty of Engineering, Universiti Teknologi Brunei, Bandar Seri Begawan, BE1410, Brunei Darussalam

^e^ Zhihai Postgraduate Working Station, Zhenjiang, Jiangsu 212000, China

*Corresponding author: [hanyongbin@njau.edu.cn](mailto:hanyongbin@njau.edu.cn) (Yongbin Han)

3 h

0 h

4 h

2 h

1 h


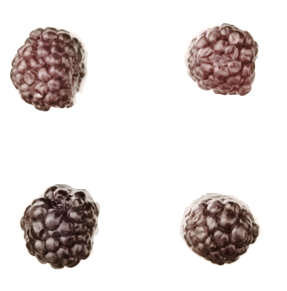

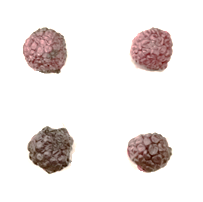

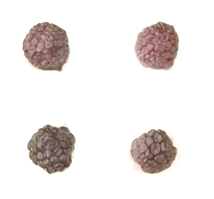

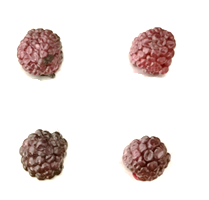

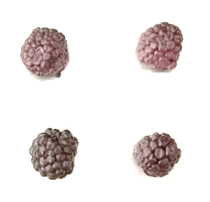


9 h


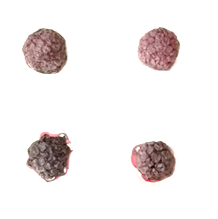


8 h

7 h

6 h

5 h


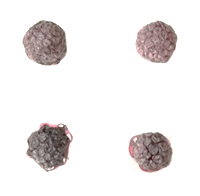

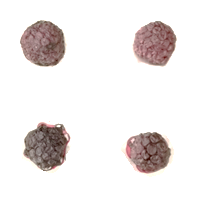

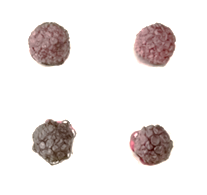

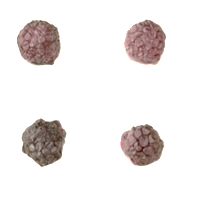


**Supplementary Fig. 1**. Images of blackberry fruit at different stages of hot air drying alone at 65 ^o^C

a


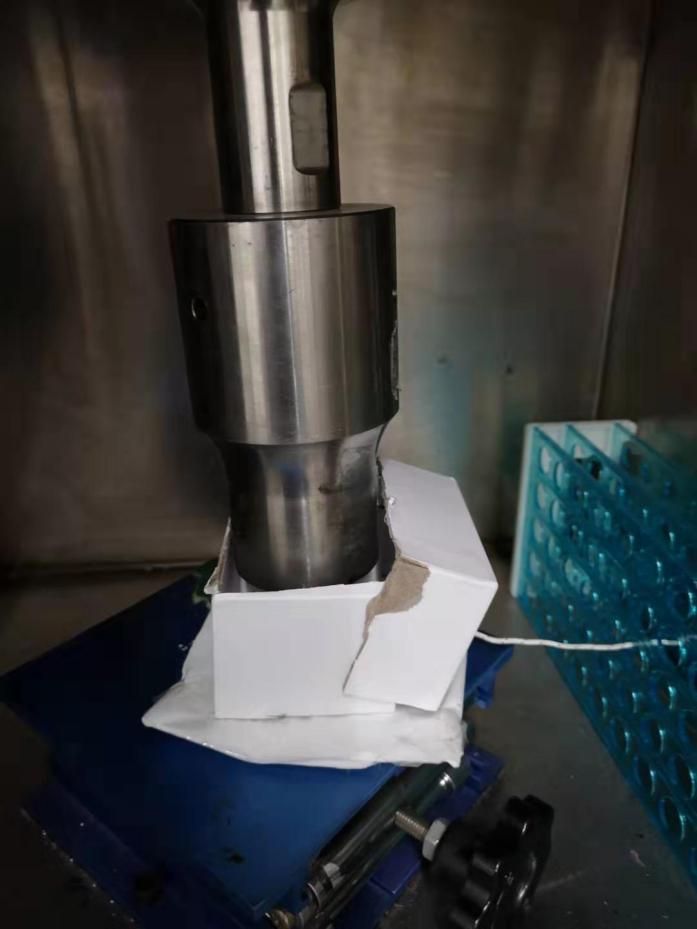


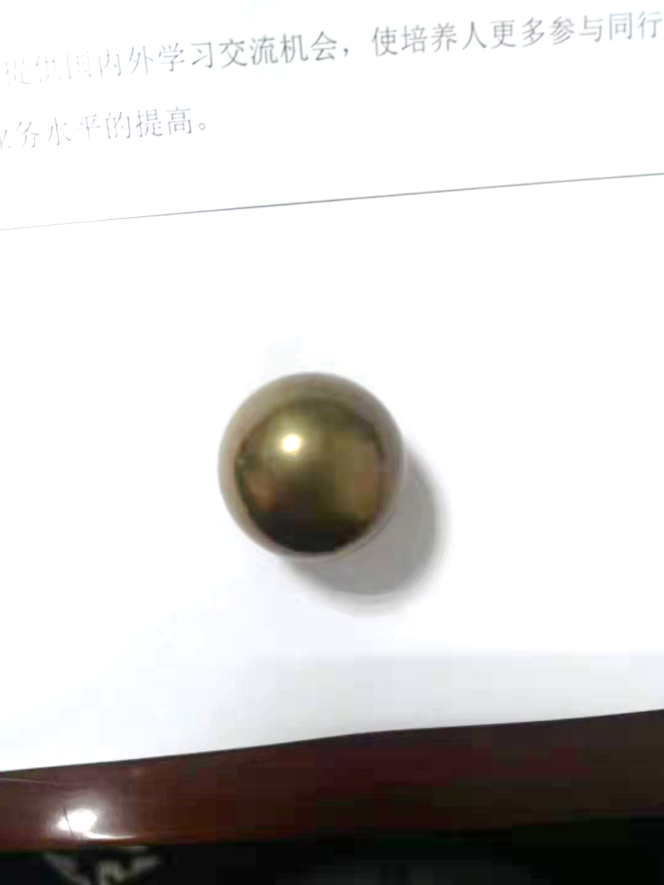


b

**Supplementary Fig. 2**. Experimental setup for the determination of actual ultrasound power for contact sonication and airborne sonication (a) and copper sphere used (b).

• Determine shrinkage pattern and heat and mass transfer path length

• Determine temperature dependent diffusivity

• Determine external heat and mass transfer coefficients

• Determine ultrasound energies dissipated on blackberry surface in the form of heat

• Build unsteady heat and mass transfer models

• Define thermophysical parameters

• Solve the models using *pdepe* function in MATLAB

• Validate the modeling results

• Display temperature and moisture distribution

**Supplementary Fig. 3**. Flow chart for numerical simulation of coupled heat and mass transfer processes under ultrasound-assisted air drying of blackberry.

a

b

c

**Supplementary Fig. 4.** Drying kinetics of blackberry sphere at 50 ^o^C (a), 60 ^o^C (b) and 70 ^o^C (c). o: air drying alone; o: air drying coupled with airborne sonication; o: air drying coupled with contact sonication.


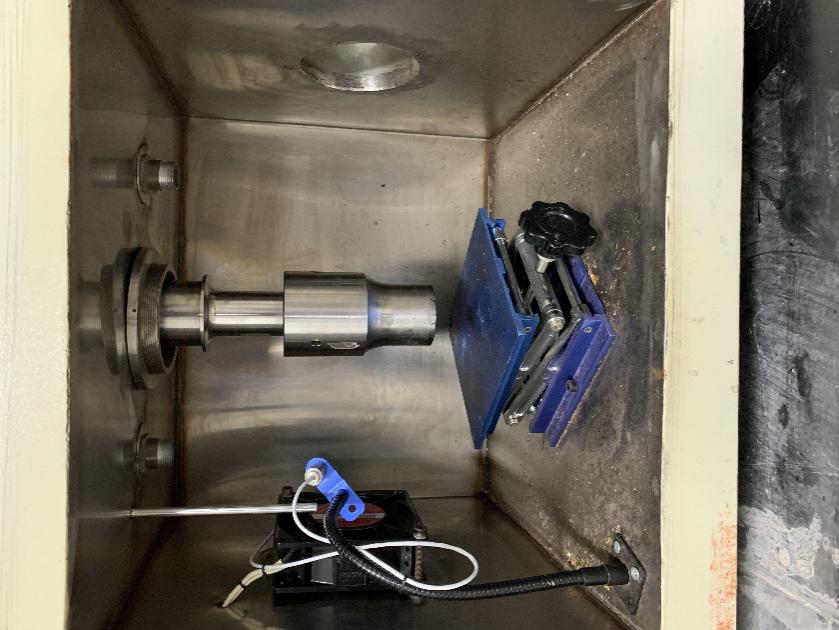


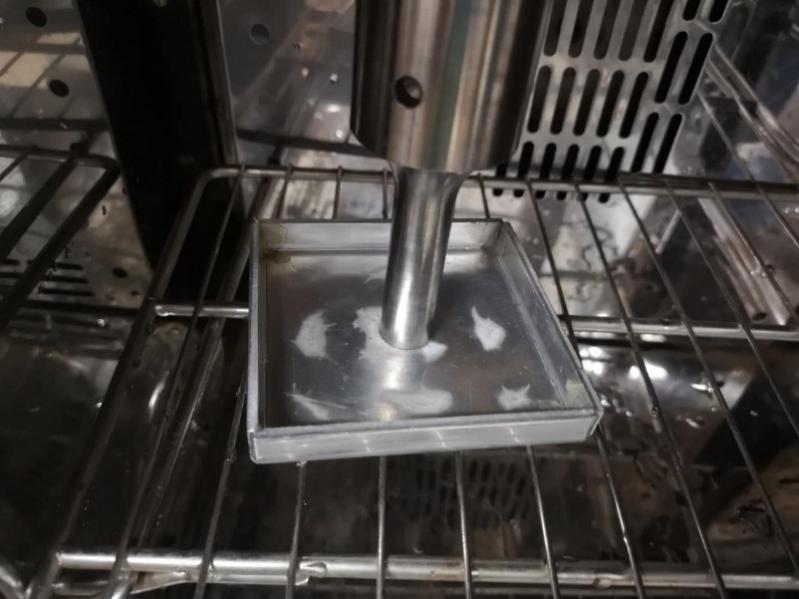


b

a


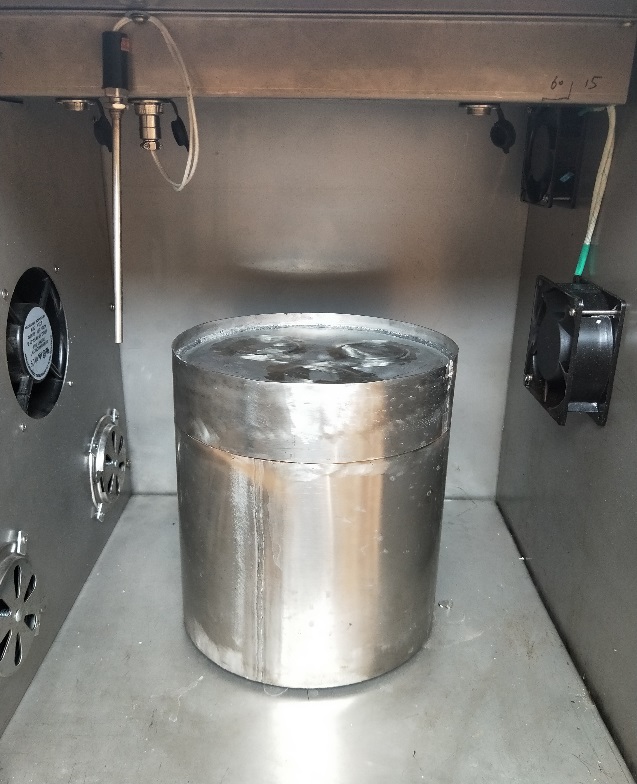

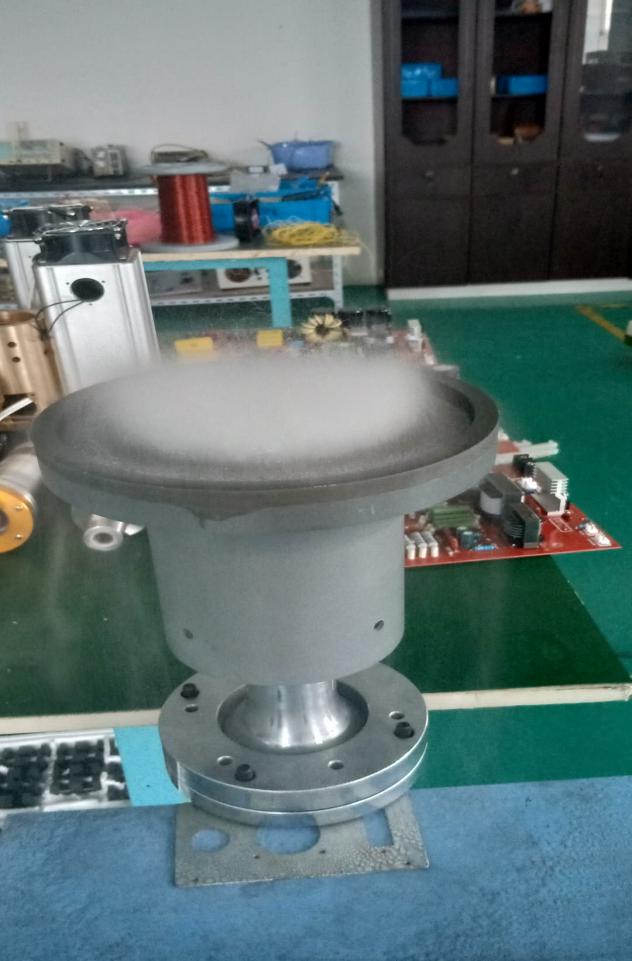


d

c

**Supplementary Fig. 5**. The ultrasound vibrators developed by the authors that can be coupled with air dryer. a: ultrasound vibrators made of stainless steel; b: ultrasound vibrators made of titanium alloy and used in this study; c: ultrasound vibrators made of aluminum alloy I; d: ultrasound vibrators made of aluminum alloy II.
